# Measurement Properties of Patient-Reported Outcome Measures for Adolescent and Young Adult Survivors of a Central Nervous System Tumor: A Systematic Review

**DOI:** 10.1089/jayao.2023.0048

**Published:** 2024-02-09

**Authors:** Kate Law, Emily Harris, Martin G. McCabe, Janelle Yorke, Sabine N. van der Veer

**Affiliations:** ^1^ Division of Nursing, Midwifery and Social Work, Faculty of Biology, Medicine and Health, The University of Manchester, Manchester, United Kingdom.; ^2^ The Christie Hospital NHS Foundation Trust, Manchester, United Kingdom.; ^3^ Division of Cancer Sciences, Faculty of Biology, Medicine and Health, The University of Manchester, Manchester, United Kingdom.; ^4^ Centre for Health Informatics, Division of Informatics, Imaging and Data Sciences, Faculty of Biology, Medicine and Health, Manchester Academic Health Science Centre, The University of Manchester, Manchester, United Kingdom.

**Keywords:** adolescent and young adult, central nervous system tumor, survivorship, patient-reported outcome measures, systematic review, validation studies

## Abstract

**Purpose:**

To identify and evaluate patient-reported outcome measures (PROMs) for assessing survivorship-related concepts for adolescent and young adult (AYA) survivors of central nervous system (CNS) tumors.

**Methods:**

We searched five electronic databases. Two researchers independently screened all titles for inclusion and used consensus-based standards for the selection of health measurement instruments (COSMIN) guidance to grade the quality of evidence for each measurement property.

**Results:**

Four studies met eligibility criteria: single-item pain thermometer; single-item fatigue thermometer; 37-item pediatric functional assessment of cancer therapy—brain tumor survivors, measuring quality of life; and 12-item Perceived Barriers Scale to assess barriers to employment. The Perceived Barrier Scale showed high-quality evidence for internal consistency and moderate quality evidence for construct and structural validity. Evidence for the measurement properties of the other PROMs was low-to-moderate quality.

**Conclusion:**

We found one PROM with sufficient evidence for good measurement properties to support its use. This warrants development and evaluation of further PROMs to inform ongoing supportive care for this population.

**Implications for Cancer Survivors:**

The Perceived Barriers Scale is sufficiently validated and could be considered to guide support for AYA survivors of CNS tumors to achieve their employment goals.

## Background

Central nervous system (CNS) tumors account for one in five childhood cancers and up to 15% of adolescent and young adult (AYA) cancers. The term AYA is commonly defined as the period between 15 and 39 years.^1^

This age range spans a period of rapid physical, cognitive, and emotional growth distinct from childhood and adulthood where individuals become independent, reach their educational and employment goals, and form lasting relationships.^2^ Immature decision-making skills, with a drive for independence, make this a unique group, with cancer during this stage disrupting the usual pattern of development with the potential to impact their future lives. Lastly, young people experience greater challenges than older patients with cancer.^3^

The AYA age range coincides with the highest rate of survival of CNS tumors.^4^ Increasing number of child and AYA CNS tumor survivors means a large proportion of patients under follow-up for CNS tumors falls within the AYA age range.^5^ Survivors of a CNS tumor are particularly at risk compared with survivors of other cancers due to the direct effect tumors can have on personality, behavior, cognition, and memory. This is in addition to other symptoms, such as altered endocrine function, pain, fatigue, seizures, depression, and sensory changes impacting vision, speech, hearing, and balance.^6–8^

The large majority of young CNS tumor survivors experience at least one ongoing symptom,^9^ and more than half experience life-altering and long-term disability.^10^ This negatively impacts their social well-being, ability to attain developmental milestones, rates of marriage and employment, and their health-related quality of life (HRQOL).^9,11,12^

Long-term follow-up services for CNS tumor survivors aim to reduce treatment sequelae and maximize opportunities to return to “normality” and reintegration into society as quickly as possible.^13^ There is increasing demand for individualized follow-up care for survivors of cancer and support with self-managing their symptoms.^14,15^ Currently, a medical model of care is still the most common method of follow-up in the United Kingdom and United States.^16^ This model tends to rate clinical care (checking for signs and symptoms of late effects) as more important than supportive care that orientates toward lifestyle advice and reintegration into education.^17^ This means many AYA CNS survivors may receive care that does not fully address their ongoing needs.

Routine use of patient-reported outcome measures (PROMs) in clinical practice has the potential to improve communication between health care professionals and patients, empower patients to report what matters to them, and overcome clinical judgments that may not truly reflect patients' circumstances.^18–20^ It can also improve symptom management, overall HRQOL, and patient satisfaction with care in people with cancer.^21,22^ Although the routine use of PROMs in oncology settings in the United Kingdom is expanding, it is not yet standard.^18,23,24^ As a result, opportunities to identify relevant patient issues, provide individualized care, and improve HRQOL are missed.

PROMs must have clinical utility, while also being valid, reliable, relevant, and acceptable to the population in which they are used.^25^ Furthermore, for PROMs to be useful for an AYA population either in a research or clinical context, they must recognize the unique needs of this age-defined population, and incorporate age-appropriate language and domain content.^26–30^ In addition to age-specific considerations, PROMs for use with survivors of a CNS tumor may require design features to decrease cognitive demand and account for cognitive and neurological disabilities, for example, by reducing complexity of items or using images instead of text.^31^

A previous review examining the use of PROMs in CNS tumor survivors identified studies in populations with a mean age >39 years,^32^ whereas reviews of PROMs focusing on pediatric CNS tumor survivors only included two studies wherein 15–18-year olds were included or wherein the mean age was between 9.5 and 13.7 years.^6,33^ These reviews also concluded that all PROMs required further evaluation of construct validity, internal consistency, and responsiveness to change. Therefore, it remains unclear which high-quality PROMs are available for the AYA CNS tumor population.

## Aim and Objectives

We explore to what extent there are PROMs available for measuring health and survivorship-related concepts in AYA survivors of CNS tumors. Ultimately, this will contribute to facilitating and improving the delivery of individualized supportive follow-up care for this patient population.

The specific objectives of our review were:

(1) Identify studies reporting the development and evaluation of PROMs to assess health and survivorship-related concepts in AYA CNS tumor survivors.
(2) For each PROM identified under 1:
  a. describe what concept it is designed to measure,
  b. describe its characteristics,
  c. assess its measurement properties, and
  d. describe its interpretability, clinical utility, and feasibility.

## Methods

We followed the 10 steps to conducting a systematic review of PROMs as proposed by the COnsensus based Standards for selection of health Measurement INstruments (COSMIN).^34^ To address objective 1, and in accordance with this guidance, steps 1–4 included formulation of the aim, formulation of eligibility criteria, literature search, and select abstracts and full text articles. For objective 2, steps 5–9 of the guidance were specific to evaluating the measurement properties of the PROM.

These steps include evaluation of content validity, evaluation of internal structure (structural validity and internal consistency), evaluation of remaining measurement properties (cross-cultural validity, reliability, measurement error, criterion validity, hypothesis testing for construct validity, and responsiveness), evaluate interpretability and feasibility, and formulate recommendations. The final step was to report the systematic review in accordance with Preferred Reporting Items for Systematic reviews and Meta-Analyses (PRISMA) reporting guidance.^35^ The study protocol was registered on the International Prospective Register of Systematic Reviews (PROSPERO; ID: 343426). No ethical approval was required to conduct this review.

### Search strategy

We searched five electronic databases, including MEDLINE, EMBASE, the Cumulative Index to Nursing and Allied Health Literature, PsycINFO, and Web of Science. The research team comprised clinical experts in the clinical specialty, experts in PROM development and evaluation, and a clinical librarian. Together, they developed the search strategy described in Table 1, incorporating terms for CNS tumors, survivors of cancer, and the PROM filter as recommended by the COSMIN guidance.^34^ In addition, the research team added search terms related to already known AYA-specific PROMs (e.g., cancer needs questionnaire)^36^ to further enable identification of evaluation studies in AYA CNS populations. We only included studies published in English without restriction of publication date.

**Table 1.** Search Terms EMBASE (OVID)

No.	Query
1	(instrumentation or methods).sh.
2	(“Validation Stud^*^” or “Comparative Stud^*^”).pt.
3	psychometry.af.
4	“psychometr^*^”.ab,ti.
5	(clinimetr^*^ or clinometr^*^).tw.
6	outcome assessment.af.
7	(“outcome assessment” or “outcome measure^*^”).ab,ti.
8	observer variation.af.
9	“observer variation”.ab,ti.
10	health status indicator.af.
11	reproducibility.af.
12	“reproducib^*^”.ab,ti.
13	discriminant analysis.af.
14	(reliab^*^ or unreliab^*^ or valid^*^ or “coefficient of variation” or coefficient or homogeneity or homogeneous or “internal consistency”).ab,ti.
15	(cronbach^*^ and (alpha or alphas)).ab,ti.
16	(item and (correlation^*^ or selection^*^ or reduction^*^)).ab,ti.
17	(agreement or precision or imprecision or “precise values”).tw.
18	(test-retest or (test and retest)).ab,ti.
19	(reliab^*^ and (test or retest)).ab,ti.
20	(stability or interrater or inter-rater or intrarater or intra-rater or intertester or inter-tester or intratester or intra-tester or interobserver or inter-observer or intraobserver or intra-observer or intertechnician or inter-technician or intratechnician or intra-technician or interexaminer or inter-examiner or intraexaminer or intra-examiner or interassay or inter-assay or intraassay or intra-assay or interindividual or inter-individual or intraindividual or intra-individual or interparticipant or inter-participant or intraparticipant or intra-participant or kappa or kappa's or kappas).ab,ti.
21	“repeatab^*^”.tw.
22	((replicab^*^ or repeated) and (measure^*^ or finding^*^ or result^*^ or test^*^)).tw.
23	(generaliza^*^ or generalisa^*^ or concordance).ab,ti.
24	(intraclass and correlation^*^).ab,ti.
25	(discriminative or “known group” or “factor analysis” or “factor analyses” or “factor structure” or “factor structures” or dimension^*^ or subscale^*^).ab,ti.
26	(“multitrait” and “scaling” and analys^*^).ab,ti.
27	(“item discriminant” or “interscale correlation^*^” or error^*^ or “individual variability” or “interval variability” or “rate variability”).ab,ti.
28	(variability and (analysis or values)).ab,ti.
29	(uncertainty and (measurement or measuring)).ab,ti.
30	(“standard error of measurement” or sensitiv^*^ or responsive^*^).ab,ti.
31	(limit and detection).ab,ti.
32	(“minimal detectable concentration” or interpretab^*^).ab,ti.
33	(“small^*^” and (real or detectable) and (change or difference)).ab,ti.
34	(“meaningful change” or “ceiling effect” or “floor effect” or “Item response model” or IRT or Rasch or “Differential item functioning” or DIF or “computer adaptive testing” or “item bank” or “cross-cultural equivalence”).ab,ti.
35	((minimal or minimally or clinical or clinically) and (important or significant or detectable) and (change or difference)).ab,ti.
36	1 or 2 or 3 or 4 or 5 or 6 or 7 or 8 or 9 or 10 or 11 or 12 or 13 or 14 or 15 or 16 or 17 or 18 or 19 or 20 or 21 or 22 or 23 or 24 or 25 or 26 or 27 or 28 or 29 or 30 or 31 or 32 or 33 or 34 or 35
37	(“patient outcome assessment^*^” or “patient reported outcome measure^*^” or “patient reported treatment outcome^*^” or “self-report^*^ measure^*^” or “self-report^*^ outcome^*^” or “patient reported outcome^*^” or “Self-Assessment”).ab,ti.
38	(“Survey” adj2 (“Health Assessment” or “Patient Assessment”)).ab,ti.
39	(“Questionnaire^*^” adj2 (“Health Assessment” or “Patient Assessment”)).ab,ti.
40	((“Self Reported” or “Patient reported”) adj3 (“recovery” or “experience” or “need^*^ assessment”)).ab,ti.
41	(PROs or PROMs or PROMIS).ab,ti.
42	(ePROs or ePROMs or ePROMIS).ab,ti.
43	exp patient-reported outcome/
44	37 or 38 or 39 or 40 or 41 or 42 or 43
45	(“living with” or “living with and beyond”).ab,ti.
46	“Post treatment”.ab,ti.
47	“Brain Tumo?r”.ab,ti.
48	(Brain adj2 (“Tumo?r” or “cancer^*^” or “malignanc^*^ neoplas^*^” or “oncolog^*^”)).ab,ti.
49	cancer survivor/
50	exp brain tumor/
51	exp survivor/
52	exp neoplasm/
53	51 and 52
54	exp “Pediatric Quality of Life Inventory”/
55	“Health Utilities Index”.mp.
56	CNQ-YP.mp.
57	MMQL-AF.mp.
58	CCSS-NAQ.mp.
59	45 or 46 or 49 or 53
60	47 or 48 or 50
61	36 or 44 or 54 or 55
62	59 and 60 and 61

To complement our electronic search, we manually searched reference lists of included studies, and conducted a targeted search in MEDLINE for any included PROMs or for comparator instruments used in their evaluation.

Eligibility criteria are described in Table 2.

**Table 2.** Inclusion and Exclusion Criteria

	Inclusion	Exclusion
Construct	Any construct related to health, illness, or support needs as assessed by patient themselves. For example, HRQOL, assessment of health care needs, physical function assessments, or more specific constructs (e.g., family functioning, reproductive need, sleep, sexual function)	Measures that exclusively reported patient-reported experience or satisfaction with health care services Any construct assessed by someone other than the person themself, e.g., proxy reports by parents^a^, teachers, or health care professionals
Population	AYAs aged 15–39 years, with a CNS tumor diagnosis, at least 6 months off treatment and with a prognosis of >12 months	Study samples wherein <50% adhered to the population criterion and/or wherein the mean age lay outside of the eligible age range; palliative patients within the last 12 months of life.
Type of instrument	Paper-based and online self-report questionnaires, including single item instruments.	Studies using structured interviews, nonquestionnaire-based tools
Study type	PROM development and validation studies wherein the aim of the study was to evaluate one or more measurement properties of the PROM and/or its feasibility, clinical utility, or interpretability. We included studies using routinely collected PROM data.	Studies wherein the PROM was used to collect outcome data rather than for PROM development or evaluation
Publication type	Studies in English published in peer-reviewed journals or full articles published in conference proceedings	Conference abstracts, nonpeer-reviewed articles, non-English articles

^a^ It is acknowledged that some survivors of a central nervous system tumor experience cognitive changes that means some may be unable to complete the questionnaire independently, although the majority of people attending follow-up are able to complete questionnaires.^36,37^ The scope of this research does not include evaluating the responses of proxy reports.

CNS, central nervous system; HRQOL, health-related quality of life; PROM, patient-reported outcome measures.

### Study selection

After removing duplicates, two researchers (K.L. and E.H.) independently screened all studies using systematic review software (RAYYAN)^38^ to record decisions. They screened all studies by title and abstract in stage 1 against the eligibility criteria (Table 2) and read full text articles where further information was required to decide on inclusion (stage 2). Reasons for exclusion were only recorded for stage 2. Any disagreements were resolved through discussion with a third member of the research team (S.N.V.D.V.).

### Data extraction

Two researchers (K.L. and E.H.) independently extracted the following data items in duplicate, resolving disagreements through discussion:

- PROM: construct, number and type of factors/domains, mode of administration, recall period, number of items, response options, interpretation of scoring, and language.
- Study population: sample size, age, gender, ethnicity, disease type, treatment, time since treatment, and country.
- Information on the methods and findings of evaluating the following measurement properties: structural validity, internal consistency, cross-cultural validity, reliability, measurement error (limits of agreement), criterion validity (correlation with gold standard), hypothesis testing, and responsiveness.
- Clinical utility and interpretability of the PROM (missing items, floor and ceiling effects, and minimal important change or minimal important difference) and its feasibility (completion time, required mental ability to complete PROM (e.g., reading age), ease of score calculation, and study setting).

### Assessing the quality of studies and evidence for measurement properties

We used the COSMIN Risk of Bias Checklist to assess the methodological quality of studies for each measurement property reported. The overall score for each study was the lowest score applied to any assessed subelement and rated as very good, adequate, doubtful, inadequate, and not applicable (N/A).^39^ We then rated the result of each study on a measurement property (except content validity and PROM development) against the COSMIN criteria for good measurement properties as sufficient (+), insufficient (−), and indeterminate (?).

We qualitatively described interpretability and feasibility data. Where results from different studies on one measurement property could be pooled, we would conduct a meta-analysis. Lastly, we used the grading of recommendations assessment, development, and evaluation to determine the quality of evidence for each measurement property.^40^

## Results

**FIG. 1.**
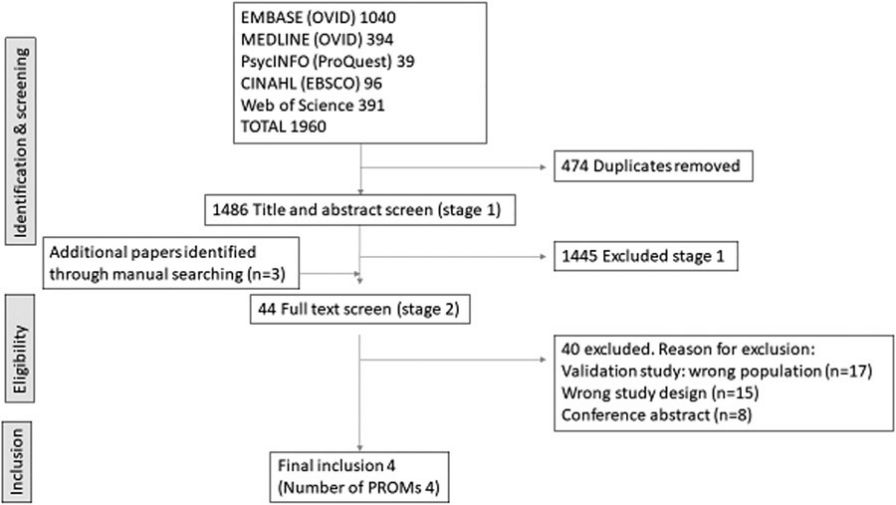
PRISMA flowchart of the screening and inclusion process (last search: May 2022). PRISMA, Preferred Reporting Items for Systematic reviews and Meta-Analyse.

After excluding duplicate titles, we screened 1486 titles and abstracts, reading the full text for 44 articles. Four studies describing four PROMs met the eligibility criteria and were evaluated and appraised in the analysis (Fig. 1). Searching reference lists of the full text articles identified a further three studies.

We excluded 17 PROMs validation studies after full text screening because they did not meet our population criteria, these are given in Table 3.

**Table 3.** Validation Studies: Reason for Exclusion

PROM	Author (year); country	Aim of study	Construct (domains)	Population characteristics			Setting
				Age in years	Disease type	Treatment type	
Excluded as mean age <15–39 years							
PedsFACT-BrS	Lai et al. (2007); United States^41^	Development of QOL measure	Quality of life (physical well-being; emotional well-being and illness experience; family and social well-being; brain tumor-specific concerns)	7–11 years (average age 9.3, SD = 1.5 part 1 item generation, average 9.8 SD = 1.3, for part 2 validation)	Range of brain tumors	Free of treatment for 1 year	Follow-up appointments
PROMIS	Lai et al. (2017); United States^42^	Evaluation of computerized adaptive testing and short forms of PROMIS	Quality of life (fatigue, mobility, upper extremity function, depressive symptoms, anxiety, peer relationships)	7–22 years mean 13.9	Range of brain tumors	Mean 3.7 (SD = 3.4) years since treatment	Follow-up appointments
PedsQL 4.0	Palmer et al. (2007); United States^43^	Reliability and validation study	Generic quality of life, Peds QL fatigue, Peds QL brain tumor module (cognitive problems, pain and hurt, movement and balance, procedural anxiety, nausea, worry)	2–18 years (average 9.76)	Range of brain tumors	34.3% off treatment >12 months, 19.2% off treatment <12 months	Completed in hospital clinic setting
HUI 2/3	Le Galès et al. (1999); France and Belgium^44^	Adapt HUI 2 and HUI 3 for use in France	Health-related quality of life (HUI 2: sensation, mobility, emotion, cognition, self-care, pain. HUI 3: vision, hearing, speech, ambulation, dexterity, emotion, cognition, pain)	8–19 years (mean age 10 boys, 12 girls)	Medulloblastoma and ependymoma	nr	Clinic settings
Excluded as mean age >15–39 years							
10-item Likert scale	Rogers et al. (2001); United States^45^	Adapting scale for brain tumor patients	Quality of life	Mean age 48 years SD = 19.87	Brain tumors	Median time since treatment 11 months	Adult brain tumor clinic
Modified SF-36	Miao et al. (2008); China^46^	To develop brain tumor-specific quality-of-life questionnaire for Chinese patients with a brain tumor	Quality of life (physiological, psychological, patient satisfaction with medical care, activities of daily living)	15–77 years, mean age 43, median 47	Glioma, meningioma, pituitary adenoma, and neurilemmoma patients	Surgery	Postoperative setting
SF-36	Bunevicius (2017); Lithuania^47^	Reliability and validity of SF-36 in patients with brain tumors	Quality of life (social functioning, general health, role limitations (physical and emotional problems), physical health, mental health, vitality, pain	Mean age 55.8 years SD 14.4	Brain tumors	nr	Approached before surgery
MDASI brain tumor module	Armstrong et al. (2006); United States^48^	Validation of MDASI brain tumor module	Symptom burden (focal symptoms, generalized symptoms, treatment, and medication-related symptoms)	18–84 years mean 45.8 (52% 18–45 years)	Range of brain tumors	On treatment. Nearly all undergone surgery, half chemotherapy or radiotherapy	Outpatient clinic
Excluded as >50% population over 39 years							
Patient concerns inventory	Rooney et al. (2014); Scotland^49^	Feasibility and clinical utility of holistic needs assessment	Patient concerns (practical, physical, family, emotional, spiritual)	18–34 years 17%, 35–59 62%, >60 years 21%	Range of brain tumors	0–12 months off treatment 47%, 53% over 13 months off treatment	Neuro-oncology outpatient clinic
Excluded as <50% diagnosed with a brain tumor							
CNQ	Clinton-McHarg et al. (2012); Australia^36^	Development of needs questionnaire	Unmet needs (treatment environment and care, feelings and relationships, daily life, information and activities, education, work)	18–23 years (median 21)	Nonhematological cancers 53% (no more detail reported)	90% off treatment (diagnosed in last 5 years)	Mailed questionnaire sent to eligible candidates
MMQL-AF	Koike et al. (2014); Japan^50^	Development of Japanese version of MMQL-AF	Quality of life (physical functioning, cognitive functioning, psychological functioning, body image, social functioning, outlook on life, human relationships)	13–19 years (mean age 15.75 SD 1.79)	3.5% had brain tumor	Off treatment at least 1 year	Eight hospitals across Japan
CCSS-NAQ	Cox et al. (2013); United States^51^	Development of comprehensive health-related needs assessment for adult survivors of childhood cancer	Unmet needs	Mean age 39 years. 25–30 13.67%, 31–41 45%	Mixed cancer types (CNS 13.41%)	Chemotherapy 24%, radiation 13%, Surgery 8%, chemo and radiation 47%, unknown 6%, no treatment 0.17%	Questionnaire mailed out
Impact of cancer	Zebrack et al. (2010); United States^52^	Psychometric validation of the impact of cancer scale for AYA survivors of childhood cancer	Impact of cancer (your body and your health, cancer treatment and health care, having children, who are you, talking and thinking about cancer, meaning of cancer, memory and thinking, finances and money, family, relationships; socializing and being with friends, life goals)	Mean 26.7 years, SD = 5.3	Mixed cancer: CNS 11.9%	Off treatment	Questionnaires mailed out
Holistic needs assessment tool (Integrated Assessment Map)	Stevens et al. (2018); United Kingdom^53^	Understanding and utilizing unmet needs of TYA's with cancer to determine priorities of care	Holistic needs assessment tool	16–24 years	Mixed cancer (7/42 had brain tumor diagnosis)	On or off treatment	Questionnaires mailed out.
Questionnaire to assess needs and experiences	Sperling et al. (2017); Denmark^54^	Development of questionnaire to evaluate treatment and survivorship from perspective of AYAs	Needs and experiences (time before treatment, being told about your illness, being a young patient, your treatment, receiving help living with and after cancer, how are you feeling today)	Phase 1 item generation: 15–29 years Phase 2 cognitive validation: 17–38 years	Phase 1: not 50% brain tumors Phase 2: no brain tumor patients included	nr	Recruited from oncology centers and cancer charity
Benefit and burden scale	Maurice-Stam et al. (2011); Holland^55^	Validation of the benefit and burden scale in young survivors of cancer in Dutch population	Impact of cancer	8–18 (mean age 13.8)	Mixed cancer types (7.8% CNS tumors)	Completed treatment 6 months–3 years previous	Questionnaires posted out
SF-SUNS	Campbell et al. (2014); Canada^56^	Development and validation of short-form SUNS	Unmet needs (financial concerns, emotional health, access and continuity of care, information, relationships)	Aged 20–39 years at diagnosis 3.7% population	Cancer survivors (“other” types made 32% including brain, gynecological, head and neck, kidney, liver, multiple myeloma)	On and off treatment	Cross-sectional survey posted out in three Canadian provinces.

AYA, adolescent and young adult; CCSS-NAQ, childhood cancer survivor study needs assessment questionnaire; CNQ, cancer needs questionnaire; HUI, health utilities index; MDASI, MD Anderson symptom inventory; MMQL-AF, Minneapolis-Manchester quality-of-life survey-adolescent form; nr, not reported; PedsFACT-BrS, pediatric functional assessment of cancer therapy-childhood brain tumor survivors; PedsQL, pediatric quality of life; PROMIS, patient reported outcome measurement information system; SF-SUNS, short-form survivor unmet needs survey; SD, standard deviation; TYA, teenage and young adult.

Included study and PROM characteristics

We included the following four PROMs:

(1) Single item screening tool for fatigue (fatigue thermometer).^57^
(2) Single item screening tool for pain (pain thermometer).^58^
(3) 12-item Perceived Barriers Scale for young people (18–30 years) to assess barriers to career development and employment.^59^
(4) 37-item quality-of-life measure (pediatric functional assessment of cancer therapy in brain tumor survivors; pediatric functional assessment of cancer therapy-childhood brain tumor survivors [PedsFACT-BrS]).^60^

The fatigue thermometer is commonly used in adult ambulatory clinics and the purpose of the study was to evaluate its use in the AYA CNS survivor population. Similarly, the pain thermometer is commonly used in children over the age of 8 years and the purpose of the study was to evaluate its use in the AYA CNS survivor population. Strauser et al.^59^ reported the development and validation of the Perceived Barriers Scale and Yoo et al.^60^ reported the validation of the PedsFACT-BrS in Korean AYA CNS survivors. Table 4 describes the PROM characteristics, as well as the characteristics of the populations in which they were developed and/or validated. All studies reported information regarding the number of items and domains where relevant.

**Table 4.** Patient-Reported Outcome Measures Characteristics and Study Population Characteristics

PROM (authors/country/year/language)	Construct	PROM characteristics							Population characteristics						
		Mode of administration	No. of factors	No. of items	Recall period	Response option	Interpretation of scoring	Response rate	Sample size	Age (years; mean)	Gender (% female)	Ethnicity (% white/Caucasian)	Disease type	Treatment	Time since treatment (years)
Fatigue thermometer (Brand et al.; United States; 2016; English)^57^	Cancer-related fatigue	nr	n/a	1	7 days	10-point Likert scale and visual analogue scale	0 no fatigue, 1–3 mild, 4–6 moderate, 7–10 severe	178/191	142	12–32 (20.24)	53	90	Low-grade glioma, embryonal tumor, germ cell, craniopharyngioma, choroid plexus, and high-grade glioma.	nr	2–27 (mean 10.55)
Pain thermometer (Chordas et al.; United States; 2013, English)^58^	Pain	nr	n/a	1	7 days	10-point Likert scale and visual analogue scale	0–3 mild pain, 4–6 moderate pain, 7–10 severe pain	116/163	99	13–32 (19.95)	52	86.8	Low-grade glioma, embryonal tumor, ependymoma, germ cell, craniopharyngioma,	nr	At least 1 year
Perceived Barriers Scale (Strauser et al.; United States; 2019; English)^59^	Perceived barriers to career development and employment	nr	Two: external barriers, internal barriers	12	nr	11-point Likert scale (0 = not a barrier, midpoint “somewhat a barrier, 10 = ‘major barrier’)”	nr	nr	110	18–30 (23.05)	52.7	90	Central nervous system tumors	Surgery, surgery/radiotherapy/chemotherapy, surgery/radiation, surgery/chemotherapy, radiation, radiation/chemotherapy, chemotherapy, stem cell transplant	At least 2 years
PedsFACT-BrS (Yoo et al.; Korea; 2010; Korean)^60^	Quality of life	nr	Four; physical, emotional, social, and familial well-being; brain tumor specific	37	4 weeks	5-point Likert scale	nr	nr	161	13–18 (15.53)	41.61	nr	Medulloblastoma, gliom, germ cell tumor, peripheral neuroendocrine tumor	Surgery, surgery/chemotherapy/radiotherapy, surgery/radiotherapy	nr

Although there was equal representation of gender identity, ethnic diversity was limited with Caucasian participants forming a majority. Chordas et al.^58^ and Strauser et al.^59^ reported participants being approached in person during clinic visit or by mail, and Yoo et al.^60^ and Brand et al.^57^ reported completion of the instruments during a clinic visit. In all studies, information was lacking on whether the PROMs were completed electronically or on paper.

### PROM measurement properties

Table 5 gives an overview of which measurement properties had been assessed for each PROM, and for each measurement property a summary of the methodological quality, the application of the criteria for good measurement properties, and the quality of evidence. Supplementary Data S1–S3 contains further details regarding the risk of bias assessment underlying our decisions on methodological quality given in Table 5.

**Table 5.** Summary of Methodological Quality and Strength of the Evidence for Each Patient-Reported Outcome Measure Property

PROM (author)	Construct	Evaluated measurement properties (+/−/?)*^a^*	Methodological quality	Summary	GRADE*^b^*			Quality of evidence for each measurement property (points downgraded)
					Risk of bias (points downgraded)	Imprecision due to sample size (points downgraded)	Indirectness (points downgraded)	
Fatigue thermometer (Brand et al.)^57^	Cancer-related fatigue	Hypothesis testing for construct validity (−)	Doubtful	Criterion validity tested but not using gold standard. Showed utility with multidimensional fatigue scale but no sensitivity and specificity.	Very serious as only one study of doubtful quality (−2)	*n* = 142 (0)	Population 12–32 years, mean age 20.24 years, SD^c^ 4.81 (0)	Low (−2)
Pain thermometer (Chordas et al.)^58^	Pain	Hypothesis testing for construct validity (−)	Doubtful	Criterion validity tested but not using gold standard. Comparator not validated. Showed utility with brief pain survey but no sensitivity and specificity.	Very serious as only one study of doubtful quality (−2)	*n* = 99 (0)^c^	Population 13–32 years, mean age 19.95 years (0)	Low (−2)
Perceived Barriers Scale (Strauser et al.)^59^	Barriers to employment and career development	Structural validity. Exploratory factor analysis demonstrates two factors account for 57% variance (+)	Adequate	Exploratory factor analysis performed, not confirmatory factor analysis	Serious as one study of adequate quality (−1)	*n* = 110 (0)	Population 18–30, mean age 23.05 years, SD 3.36 (0)	Moderate (−1)
		Internal consistency, Cronbach α > 0.7 (+)	Very good	Only relevant if based on reflexive model—unable to determine whether Perceived Barriers Scale is based on reflexive model.	No risk of bias (0)			High
		Hypothesis testing for construct validity (+)	Adequate	Comparator instruments not tested in same populations	Serious as one study of adequate quality (−1)			Moderate (−1)
PedsFACT BrS (Yoo et al.)^60^	Quality of life	Internal consistency Cronbach α > 0.7 (+)	Very good	Cronbach alpha's calculated, all >0.7	No risk of bias (0)	*n* = 161 (0)	Population 13–18 years, mean age 15.53 years, SD 1.95 (−1)	Moderate (−1)
		Reliability (internal consistency coefficients) Cronbach α 0.81 and 0.94 (+)	Adequate	Assumable patients were stable in time interval, no information reported.	Serious (−1)			Low (−2)
		Hypothesis testing for construct validity (+)	Adequate	Comparator instrument validated in a younger population (mean age 9.8 years) and only 7% of this population had a brain tumor (Lai et al., 2007). Testing only for correlates for anxiety and depression not all areas of Quality of Life	Serious (−1)			Low (−2)
		Hypothesis testing for known group validity (+)	Very good	Karnofsky scores, treatment types and treatment status described clearly. Correlations as expected.	No risk of bias (0)			Moderate (−1)

^a^ Measurement properties rated as sufficient (+), insufficient (−), indeterminate (?).

^b^ Per PROM, there was only one evaluation per measurement property, “inconsistency” did not apply. We, therefore, used the modified GRADE approach to grade the quality of evidence.

^c^ As the sample size was only one participant below the recommended threshold of 100 for downgrading, we did not downgrade Chordas et al.

COSMIN, consensus-based standards for the selection of health measurement instruments; GRADE, the grading of recommendations of assessment, development, and evaluation.

The most common measurement property evaluated for all four PROMs was hypothesis testing for construct validity: that is, testing the assumption that the PROM validly measures the construct it aims to assess. We graded the quality of evidence for this measurement property in all studies as low, except for the Perceived Barrier Scale, where it was moderate. As each PROM was measuring a different construct, it was not possible to perform a meta-analysis. We graded the quality of evidence for internal consistency of the Perceived Barriers Scale and PedsFACT-BrS as high and moderate, respectively, with both instruments demonstrating good correlation between items. Lastly, Strauser et al.^59^ reported PROM development in collaboration with users and health professionals, suggesting content validity of the Perceived Barriers Scale.

Risk of bias in PROM development and content validity is assessed using COSMIN checklist though data are not analyzed and given a score on methodological quality. Information regarding the development of the Perceived Barriers Scale included interviews with patients and health care professionals to maximize comprehensiveness and content validity.^59^ The translated PedsFACT-BrS was also pretested with young people to assess comprehensiveness.^60^ Despite content validity being considered the most important measurement property,^39^ limited information as reported in these articles meant much of the checklist could not be completed and no other published studies were found to report on the development of the PROMs.

### PROM interpretability, clinical utility, and feasibility

Information on interpretability or clinical utility was lacking in all studies. Regarding feasibility, all studies except Strauser et al.^59^ reported information on missing data. Yoo et al.^60^ reported that they imputed missing data using the mean score for the scale or subscale where data were missing and no >20% of items within a subscale were missing. Chordas et al.^58^ and Brand et al.^57^ excluded incomplete data sets from their analysis.

No studies reported floor/ceiling effects or information on what they considered a minimal important difference for their PROM. Data collection for all instruments happened in the follow-up clinic setting or through mailshot, but only Yoo et al.^60^ reported the time taken to complete the instrument (15–20 minutes). Supplementary Data S3 provides further details on what studies reported on PROM interpretability, clinical utility, and feasibility.

## Discussion

Our review identified four PROMs developed and evaluated to assess survivor-related concepts in AYA CNS tumor survivors. The Perceived Barriers Scale had moderate-to-high quality evidence for sufficient internal consistency and structural and construct validity. This PROM may, therefore, be suitable for use in AYA CNS survivors, and could help patients to achieve their employment goals and contribute to increasing their (currently low) rates of employment.^61,62^ The study evaluating the PedsFACT-BrS found that the quality-of-life instrument had sufficient internal consistency, reliability, and construct and known group validity.

However, the quality of the underlying evidence for these measurement properties was low to moderate, warranting further evaluation studies before the PedsFACT-BrS can be recommended for use in practice for this specific population.

AYA CNS tumor survivors often experience multiple long-term conditions that affect their independence and HRQOL. Once further evidence for sufficient measurement properties is available, the PedsFACT-BrS, or similar instruments, could facilitate monitoring of HRQOL and inform follow-up services to optimize HRQOL. The two single-item screening tools (i.e., the fatigue thermometer and the pain thermometer) had low-quality evidence for insufficient measurement properties and can, therefore, not be recommended for use. Lastly, future research should assess cross-cultural validity, measurement error, and responsiveness, which were not evaluated in any of the studies. This is in keeping with findings from other PROM reviews in adult^32^ and pediatric CNS cancer populations.^6,33^

The COSMIN guidance advises only including studies wherein at least 50% of participants meet the eligibility criteria for the review. To adhere to this guidance, we excluded several PROM validation studies wherein at least half of their study population did not consist of AYA CNS survivors, or, wherein insufficient information to assess the population characteristics was reported. Lack of such information hampers making robust recommendations on whether a PROM is suitable for this group. This is supported by multiple studies highlighting that PROMs data can be inaccurate or inadequate if the PROM does not contain relevant age-appropriate domains representative of the target disease population^26,63–67^

Researchers should, therefore, report complete and sufficiently detailed information about the age and conditions of their study population. This will help maximize the utility of future research on PROMs for AYAs with a specific type of cancer, particularly since individual studies for this group are typically relatively small. In addition, detailed population characteristics facilitate assessing indirectness when grading the quality of evidence. For example, for Yoo et al.,^60^ the mean age of participants met our population inclusion criteria but the reported age range suggested that their study participants might not fully reflect our target population.

### Limitations

There is a risk of publication bias in our review because PROMs evaluation studies reporting negative results may not have been published. This means we may have missed studies of PROMs for this unique population. In addition, there may be language bias as we only included publications in English.

We complemented our search terms with the names of PROMs that we knew were available for mixed AYA cancer populations. PROMs not known to our multidisciplinary research team were, therefore, less likely to be picked up by our search strategy.

Lastly, our review aimed to identify PROMs measuring any construct, despite COSMIN advising a PROMs review to ideally focus on one construct only.^39^ It is well known that survivors of CNS tumors may experience multiple ongoing issues, and any validated PROM could be used in follow-up services to enhance the support offered. Combined with the anticipated scarcity of PROMs studies for AYA CNS tumor survivors, we, therefore, deemed it appropriate to include all constructs in one review.

### Implications for clinical practice and research

Our review highlighted attempts to assess fatigue, pain, and barriers to employment, all of which are reported as factors in the lives of CNS tumor survivors that may impact patients' HRQOL and their ability to reach independence.^68–70^ The implementation of tools that identify ongoing issues is advocated and would facilitate provision of personalized follow-up,^14,15^ as well as clinical audits and service evaluations.^71^ However, the routine use of such tools is still largely lacking in follow-up services.

This review suggests that services could consider the Perceived Barriers Scale to identify barriers to employment and identify and address individual needs. However, for this PROM to support audit and service evaluation, further research should examine its reliability and responsiveness to change. The PedsFACT-BrS also showed several sufficient measurement properties for evaluating HRQOL in this population, but further research should strengthen the current evidence base, while also evaluating the instrument's responsiveness to change.

The use of reliable tools can allow future research to examine the effectiveness of interventions designed to lessen unmet needs by measuring and quantifying changes. Future studies may also focus on evaluating PROMs for AYA survivors of CNS tumors that were originally developed and validated in populations with mixed cancer types or older/younger populations.

Lastly, given that a PROM can measure any aspect of survivorship and survivors of a CNS tumor experience many ongoing symptoms, it must be acknowledged that we are at risk of burdening patients with multiple PROMs designed to measure different aspects of survivorship. Furthermore, some may perceive the use of PROMs as impersonal and negatively impacting the patient–clinician relationship.^72^

Future research may, therefore, also consider examining the preference of how young people would like unmet needs to be assessed and how they can be used without detriment to the patient–professional relationship.

## Conclusion

We found four PROMs for assessing survivorship-related concepts in AYA survivors of CNS tumors, but only the Perceived Barriers Scale had sufficient quality evidence for its measurement properties. In the absence of any other validated instruments to assess barriers to employment, this scale could be considered for use in practice, although further research is necessary to evaluate its reliability and responsiveness to change. Our review warrants development and evaluation of additional PROMs to inform supportive care for this population if we are to address the complex ongoing sequelae and provide personalized support directed to those AYA survivors of CNS tumors in need.

## Supplementary Material

Supplemental data

Supplemental data

Supplemental data
