# Supplementary material for: Measurement Properties of Patient-Reported Outcome Measures for Adolescent and Young Adult Survivors of a Central Nervous System Tumor: A Systematic Review
Source: J Adolesc Young Adult Oncol. 2024 Feb 9;13(1):40–54. doi: 10.1089/jayao.2023.0048 (PMC10877386; doi:10.1089/jayao.2023.0048)
Supplement: Supplemental data [file Supp_DataS1.docx]

Supplementary material.1.

Summary of methodological quality (risk of bias) and criteria for good measurement properties (no information on cross cultural validity, measurement error, criterion validity and responsiveness therefore these measurement properties are excluded from table)

| \| Concept \| PROM (Author/ Country/ Year) \| INTERNAL VALIDITY \| \| REMAINING MEASUREMENT PROPERTIES \| \| \| \| --- \| --- \| --- \| --- \| --- \| --- \| --- \| \| Structural validity \| Internal consistency \| Reliability \| Hypothesis testing (construct validity: construct validity \| Hypothesis testing: comparison between subgroups (discriminative/ known group) \| \| \| Cancer related fatigue \| Fatigue Thermometer (Brand et al/ USA/ 2016) [57] \|  \|  \|  \| n=142. Methodological quality= **Doubtful**. Results: **-** as no cutoff met apriori criteria for acceptable sensitivity (≥0.90) and specificity (≥0.75) despite showing good correlation with Multi-dimensional fatigue scale (area under the curve 0.822). \|  \| \| Pain \| Pain thermometer (Chordas et al/ USA/ 2013) [58] \|  \|  \|  \| n=99. Methodological quality= **Doubtful**. Results: - no cutoff met apriori criteria (sensitivity >0.90, specificity >0.75). Good correlation with Brief Pain Survey (area under the curve 0.828). \|  \| \| Career development and employment \| Perceived barriers scale (Strauser et al/ USA/ 2019) [59] \| n=110. Methodology quality= **adequate**. Results: **+** \| n=110. Methodology quality= **very good**. Results: **+** as crohnbach alpha >0.70: Internal barrier 0.88; external barrier 0.80. \|  \| n=110. Methodological quality= **adequate**. Results: **+** as hypothesis supported. \|  \| \| Quality of life \| PedsFACT-BrS (Yoo et al/ Korea/ 2010) [60] \|  \| n=161. Methodological quality= **very good**. Results: **+** crohnbach alpha >0.70 \| N=161, methodological quality= **adequate**. Results **+** correlation coefficient 0.81 for social and family subscale and 0.94 for physical well-being subscale**.** \| N=161, Methodological quality= **adequate**. Results: **+** hypothesised that EWB would be correlated with depression and anxiety is supported. Divergent validity seen between social well-being and physical well-being as hypothesised. \| n=161, Methodological quality= **very good**. Results: **+** results enabled discrimination by karnofsky score (100,90 or 80). Analysis by treatment type showed discrimination in physical well-being(p<0.05), EWB (p<0.05) and overall (p<0.05). Significant difference seen between those on/off treatment only seen in physical well-being scale (p<0.001). \| |  |  | |  | | |
| --- | --- | --- | --- | --- | --- | --- | --- | --- | --- | --- | --- | --- | --- | --- | --- | --- | --- | --- | --- | --- | --- | --- | --- | --- | --- | --- | --- | --- | --- | --- | --- | --- | --- | --- | --- | --- | --- | --- | --- | --- | --- | --- | --- | --- | --- | --- |
|  |  |  |  |  |  |  |
|  |  |  |  |  |  |  |
|  |  |  |  |  |  |  |
|  |  |  |  |  |  |  |
|  |  |  |  |  |  |  |
|  |  |  |  |  |  |  |
