# Supplementary material for: Measurement Properties of Patient-Reported Outcome Measures for Adolescent and Young Adult Survivors of a Central Nervous System Tumor: A Systematic Review
Source: J Adolesc Young Adult Oncol. 2024 Feb 9;13(1):40–54. doi: 10.1089/jayao.2023.0048 (PMC10877386; doi:10.1089/jayao.2023.0048)
Supplement: Supplemental data [file Supp_DataS3.docx]

Supplementary material. 3

Interpretability, clinical utility and feasibility

| PROM | Author (Country), year | Purpose | Interpretability | Feasibility | | |
| --- | --- | --- | --- | --- | --- | --- |
|  |  |  | Missing data | Length of instrument | Completion time | Study setting |
| fatigue screening tool | Brand et al (USA) 2016^57^ | screening tool for use in routine care | 36 excluded due to missing data | 1 item | nr | multi-disciplinary survivor-ship visit |
| Pain screening tool | Chordas et al (USA) 2013^58^ | screening tool for use in routine care | 17 incomplete | 1 item | nr | Neuro-oncology outcomes clinic |
| Perceived barriers scale | Strauser et al (USA) 2018^59^ | identify barriers to career development and employment in clinical and research setting | nr | 12 items | nr | long-term follow-up care clinic |
| PedsFACT-BrS | Yoo et al (Korea) 2010^60^ | Quality of life measure | 1.8% | 37 items | 15-20 minutes | routine follow-up clinic (multi-centre) or posted home |

nr= Not reported. USA= United States of America
Information on interpretability (distribution of scores in study population; floor/ceiling effects; minimal important difference) was not reported.
Information on ease of administration, ability required to complete PROM or ease of scoring was not reported.
