# Supplementary material for: Measurement Properties of Patient-Reported Outcome Measures for Adolescent and Young Adult Survivors of a Central Nervous System Tumor: A Systematic Review
Source: J Adolesc Young Adult Oncol. 2024 Feb 9;13(1):40–54. doi: 10.1089/jayao.2023.0048 (PMC10877386; doi:10.1089/jayao.2023.0048)
Supplement: Supplemental data [file Supp_DataS2.docx]

Supplementary material. 2.

Evidence for decision regarding methodological quality: risk of bias in: Fatigue Thermometer.^57^

| **Measurement property** | **COSMIN Risk of bias Standard** | **Data** | **Score** |
| --- | --- | --- | --- |
| **Box 9a. Hypothesis testing: construct validity (comparison with existing outcome measures, convergent validity)** | Is it clear what the comparator instruments measure | PedQoL multidimensional fatigue scale, 18 item likert scale. Higher MFS=better functioning and fewer fatigue symptoms. Based on 3 subscales, the measure provides a score based on each subscale and an overall fatigue score. These measured the same construct. | Very Good |
|  | Were measurement properties of the comparator instrument sufficient | Internal consistency of MFS evaluated using Crohnbach α: between 0.87 and 0.94 for each subscale of MFS. Known group hypothesis testing evaluated construct validity using t-test and ANOVA between healthy children and children with cancer. MFS validated in 2-18yr olds with cancer, only 7% of the population had a brain tumour. Hypothesis testing for construct validity also tested by evaluating correlations between MFS and the PedQoL generic scale. Hypothesis confirmed healthy children score higher, meaning better HRQoL and less fatigue. Validated in patients with mixed cancer types.^73^ | Adequate |
|  | Were design and statistical methods adequate for the hypothesis to be tested | Pearson correlation not reported. | Doubtful |
|  | Any other flaws | Correlations not reported for comparison between the tests, however results reported indicate criterion validity has been tested: Concordance sensitivity and specificity values of each subscale are reported, examined using ROC and AUC. ROC used to examine sensitivity and specificity. AUC used to quantify the diagnostic utility of the FT. Sensitivity and specificity values are reported for selected cutoff scores. Cutoff scores set for sensitivity (>0.9) and specificity (>0.75). IF the MFS was true gold standard, ROB=Very Good. | Doubtful |

Pain Thermometer^58^

| **Measurement property** | **COSMIN ROB standard** | **Data** | **Score** |
| --- | --- | --- | --- |
|  |  |  |  |
| **Box 9a. Hypothesis testing: construct validity (comparison with existing outcome measures, convergent validity)** | Is it clear what the comparator instruments measure | Brief Pain Survey, 15 items, modelled from the Brief Pain Inventory. Contains 2 items on pain severity, 2 items on pain interference, 3 items asking how many days they experienced no pain/moderate pain/severe pain. 4 items about pain attribution and 3 regarding pain medication. Recall 1 wk. | Very good |
|  | were measurement properties of the comparator instrument sufficient | BPS not validated, but based on Brief Pain Inventory which is validated in adult survivors of cancer.^74^ However internal consistency of 6 items measuring pain severity, interference and frequency indicated good consistency (crohnbach α 0.81). | Inadequate |
|  | was statistical method appropriate | correlations not reported. | Doubtful |
|  | any other flaws | results reported are as if the BPS was a gold standard and criterion validity was evaluated: ROC and AUC reported. If the PT showed specificity AUC >0.75 (indicative of good discrimmination) and Sensitivity >0.9 could be recommended for routine use. Agreement quantified by calculating the sensitivity of PT to accurately identify individuals who have pain by producing a positive screening result. Specificty of PT quantified by correctly identifying those who do not have pain and have a negative PT result. Sensitivity and specificity values are reported for selected cutoff scores. Cutoff scores set for sensitivity (>0.9) and specificity (>0.75). If criterion validity was assessed, ROB=Very good. | Doubtful |

Perceived Barriers Scale^59^

| **Measurement property** | **COSMIN ROB standard** | **Data** | **Score** |
| --- | --- | --- | --- |
| **Box 3: structural validity (if reflexive model)** | For CTT: Was exploratory or confirmatory factor analysis performed? | Exploratory (not confirmatory) factor analysis performed and identified two factors (internal and external barriers to employment and career devel). | Adequate |
|  | or IRT/Rasch: does the chosen model fit to the research question? | *(IRT the measurement precision depends on the latent-attribute value. CTT one uses common estimate of the measurment precision assumed to be equal for all individuals irrespective of attribute levels).* |  |
|  | Was the sample size included in the analysis adequate? | >7 times per item | Very Good |
|  | any flaws | Unable to determine if the instrument is based on a reflexive model. The items appear to be causal indicators, therefore according to COSMIN guidance, structural validity cannot be measured as items will not be related to each other as in reflexive models (where items are effect indicators). COSMIN recommend to report these results where they have been published and if it is unclear whether the model is causative or reflexive. |  |
| **Box 4: Internal consistency (is it based on reflexive model?)** | Was an internal consistency statistic calculated for each unidimensional scale or subscale separately? | crohnbach alpha reported for each subscale. | Very Good |
|  | For continuous scores: Was Cronbach’s alpha or omega calculated? | yes | Very Good |
|  | For dichotomous scores: Was Cronbach’s alpha or KR‐ 20 calculated | Not applicable | _ |
|  | For IRT‐based scores: Was standard error of the theta (SE (θ)) or reliability coefficient of estimated latent trait value (index of (subject or item) separation) calculated? | Not applicable | _ |
|  | any other flaws | Same as above regarding definition of reflexive/ formative model. |  |
| **Box 9: hypoth testing: construct validity** | Is it clear what the comparator instrument(s) measure(s) | CSE and WHODAS | Very Good |
|  | Were the measurement properties of the comparator instrument(s) sufficient? | Yes, internal consistency >0.7 for both. Test-retest reliability 0.81 for CSE. However, they weren't validated in same populations: WHODAS validated in people with Huntington Disease, CSE validated in two sets of employers in two different companies and two universities. | Adequate |
|  | Was the statistical method appropriate for the hypotheses to be tested? | Pearson product calculated for each item against the PBS. | Very Good |

PedsFACT –BrS^60^

| **Measurement property** | **COSMIN ROB standard** | **Data** | **Score** |
| --- | --- | --- | --- |
| **Box 4: internal consistency** | Was an internal consistency statistic calculated for each unidimensional scale or subscale separately? | Crohnbach α calculated for each subscale | Very Good |
|  | For continuous scores: Was Cronbach’s alpha or omega calculated? | Crohnbach α | Very Good |
|  | For dichotomous scores: Was Cronbach’s alpha or KR‐ 20 calculated | Not applicable |  |
|  | For IRT‐based scores: Was standard error of the theta (SE (θ)) or reliability coefficient of estimated latent trait value (index of (subject or item) separation) calculated? | Not applicable |  |
|  | any other flaws | but qol is a causative model so not required to test internal consistency |  |
| **Box 6: reliability** | were patients stable in interim period | no information reported, assumable patients were stable | Adequate |
|  | were time intervals appropriate | 7-10 days | Very Good |
|  | were test conditions similar | information not reported, assumable conditions were same | Adequate |
|  | for cont scores: was ICC calculated | Internal consistency coefficient calculated, no formula provided | Adequate |
|  | for dichot scores: was kappa coefficient calculated | Not applicable |  |
|  | for ordinal: was weighted kappa coefficient calculated | Not applicable |  |
|  | for ordinal: was weighting scheme described? |  |  |
|  | any flaws? | Only 30 at time 2. |  |
| **Box 9a Hyptohesis testing: construct validity** | Is it clear what the comparator instrument(s) measure(s) | Revised Childrens Manifest Anxiety Scale 37 items. Kovacs Childrens Depression Inventory.^75^ | Very Good |
|  | Were the measurement properties of the comparator instrument(s) sufficient? | Crohnbach 0.81 with RCMAS and 0.72 with CDI. (information given on both questionnaires therefore analyses considered as one study). Comparator not validated in same population. | Adequate |
|  | Was the statistical method appriopriate for the hypotheses to be tested? | Pearson product scores used to determine convergent and divergent validity | Very Good |
| **Box 9b: hypothesis testing: known groups** | Was an adequate description provided of important characteristics of the subgroups? | 3 subgroups: Karnofsky scores of 100, 90 or 80. On/off treatment and type of treatment. | Very Good |
|  | Was the statistical method appropriate for the hypotheses to be tested? | ANOVA and t-test to discrimminate across Karnofsky score, type of treatment and receiving treatment status. | Very Good |
|  | Were there any other important flaws in the design or statistical methods of the study? |  |  |
